# Impact of climate warming on soil microbial communities during the restoration of the inner Mongolian desert steppe

**DOI:** 10.3389/fmicb.2024.1458777

**Published:** 2024-09-06

**Authors:** Jirong Qiao, Jiahua Zheng, Shaoyu Li, Feng Zhang, Bin Zhang, Mengli Zhao

**Affiliations:** Key Laboratory of Grassland Resources of the Ministry of Education, Key Laboratory of Forage Cultivation, Processing and High Efficient Utilization of the Ministry of Agriculture and Rural Affairs, Inner Mongolia Key Laboratory of Grassland Management and Utilization, College of Grassland, Resources and Environment, Inner Mongolia Agricultural University, Hohhot, China

**Keywords:** climate change, degraded grassland restoration, soil microbial diversity, microbial network complexity, microbial network stability

## Abstract

**Introduction:**

Grazer exclosure is widely regarded as an effective measure for restoring degraded grasslands, having positive effects on soil microbial diversity. The Intergovernmental Panel on Climate Change (IPCC) predicts that global surface temperatures will increase by 1.5–4.5°C by the end of the 21st century, which may affect restoration practices for degraded grasslands. This inevitability highlights the urgent need to study the effect of temperature on grassland soil microbial communities, given their critical ecological functions.

**Methods:**

Here, we assessed the effects of heavy grazing (control), grazer exclosure, and grazer exclosure plus warming by 1.5°C on soil microbial community diversity and network properties as well as their relationships to soil physicochemical properties.

**Results and discussion:**

Our results showed that grazer closure increased soil microbial richness relative to heavy grazing controls. Specifically, bacterial richness increased by 7.9%, fungal richness increased by 20.2%, and the number of fungal network nodes and edges increased without altering network complexity and stability. By contrast, grazer exclosure plus warming decreased bacterial richness by 9.2% and network complexity by 12.4% compared to heavy grazing controls, while increasing fungal network complexity by 25.8%. Grazer exclosure without warming increased soil ammonium nitrogen content, while warming increased soil nitrate nitrogen content. Soil pH and organic carbon were not affected by either exclosure strategy, but nitrate nitrogen was the dominant soil factor explaining changes in bacterial communities.

**Conclusion:**

Our findings show that grazer exclosure increases soil microbial diversity which are effective soil restoration measures for degraded desert steppe, but this effect is weakened under warming conditions. Thus, global climate change should be considered when formulating restoration measures for degraded grasslands.

## 1 Introduction

Soil microbial communities play a critical role in maintaining the functioning of grassland ecosystems (Liu et al., 2020; Coban et al., 2022). These microbes facilitate plant and soil restoration by influencing litter decomposition, organic substrate transformation, and mineral nutrient supply (Lindsay and Cunningham, 2009; Coban et al., 2022; Pedrinho et al., 2024), with bacteria and fungi dominating these processes due to their high biodiversity, complex taxonomic composition, and ubiquitous influence on biogeochemical cycles (Zhou et al., 2023). However, their diversity, species composition, and network properties face serious threats due to human activities and global climate change (Pedrinho et al., 2024). Grazing is the traditional method of grassland management in Inner Mongolia; specifically, the desert steppe ecosystem is ecologically fragile compared to other grasslands, and its microbial diversity is very sensitive to grazing disturbance (Zhang et al., 2021). In recent years, overgrazing and continuous climate warming have escalated ecological challenges to the desert steppe, making the restoration of degraded grasslands a pressing issue (Zhou et al., 2010; Wu Y. et al., 2022; He et al., 2022). Grazer exclosure is a widely employed grassland management method for restoring degraded grasslands, by introducing a physical barrier against unwanted grazing animals (Medina-Roldán et al., 2012; Tang et al., 2016; Yao et al., 2018; Zhang M. X. et al., 2023). Therefore, studying the effects of grazer exclosure on soil microbial diversity, especially fungal and bacterial diversity, under global climate change will help improve the diversity and restoration of degraded grasslands.

Grazing affects soil microbial community composition and diversity primarily through livestock excreta deposition and trampling (Yang et al., 2019), and grazer exclosure might mitigate these grazing effects (Fan et al., 2020). Previous studies have shown that compared to free-grazed areas, grazer exclosure promotes the colonization of eutrophic microbial taxa (Cao et al., 2022), alters soil microbial community composition (Yang et al., 2019), and increases microbial richness and soil network connectivity (Morriën et al., 2017). However, long-term grazer exclosure may harm soil microbial network stability (Chen et al., 2023). Several studies have shown that soil moisture, pH, and nutrients (including organic carbon and nitrogen) are key drivers of microbial composition and structure (Lauber et al., 2009; Yao et al., 2018; Wang et al., 2019). Specifically, microbial communities tend to be more richly structured under higher soil moisture and nutrient content, with bacterial diversity greater in neutral soils than in acidic ones (Kang et al., 2021; Philippot et al., 2024). Grazer exclosure significantly increases aboveground vegetation cover (Yang et al., 2016) and the abundance of plant-derived food resources (e.g., leaf litter and roots) entering the soil (Chen and Chen, 2018). This leads to higher soil moisture and nutrient levels (Chen et al., 2023), particularly organic carbon content, which, in turn, enhances microbial diversity and network stability (Kang et al., 2021; Wu et al., 2021). Additionally, the concentration of nitrate nitrogen is crucial in shaping bacterial and fungal communities (Wang et al., 2019; Zhang et al., 2024). Grazer exclosure reduces the input of livestock waste and consequently affects the effectiveness and distribution of soil nitrogen, which leads to changes in microbial composition. Importantly, the IPCC predicts global surface temperatures will increase between 1.5 and 4.5°C by the end of the 21st century (IPCC, 2013). According to the metabolic theory of ecology (MTE), higher temperatures will stimulate interactions among various species, leading to the formation of more complex microbial networks (Brown et al., 2004; Guo et al., 2019; Yuan et al., 2021). However, rising temperatures are also expected to reduce soil microbial alpha diversity and alter beta diversity (Wu L. et al., 2022; Zhao et al., 2024). Previous studies have shown that the impact of global warming on the soil microbial community is mediated by changes in soil water availability and nutrient availability (Weedon et al., 2012; Zhang et al., 2015), largely, by changes in soil nitrogen availability (Li et al., 2024).

Taken together, both grazer exclosure and warming have varying impacts on soil microbial communities. However, the influence of global warming on the recovery processes of these communities under grazer exclosure remains unclear. Understanding this will aid in the development of effective grassland management and conservation strategies to mitigate the effects of climate change (Guo et al., 2019). Therefore, this study investigates changes in soil microbial alpha diversity, beta diversity, network complexity, and network stability in the desert steppe under three conditions: free grazing (CK), grazer exclosure (GE), and grazer exclosure plus warming by 1.5°C (GE + W). We tested two hypotheses: (1) Grazer exclosure normally promotes soil microbial restoration, but this effect is limited under warming conditions, and (2) warming-induced changes in soil nutrient content are a key factor limiting the recovery of soil microbial communities in degraded arid and semi-arid grassland ecosystems.

## 2 Materials and methods

### 2.1 Study sites and experimental design

The experimental site is located in the desert steppe of Siziwangqi (41°46′43′′ N, 111°53′42′′ E, at 1450 m above sea level), Inner Mongolia, China. This region is characterized by aridity and low rainfall, with an average annual temperature of 4.15°C, mean annual precipitation of 229 mm, and sandy loam soil (FAO soil classification). This area is mainly used for grazing, and the dominant plant species are *Stipa breviflora*, *Cleistogenes songorica*, and *Artemisia frigida* (Zhang F. et al., 2023).

The experiments were conducted on a long-term (20-year) grazing platform that included no grazing, light grazing, moderate grazing, and heavy grazing treatment. The stocking rates were based on the results of Wei et al. (2000) with 0, 0.91, 1.82, and 2.71 sheep ha^−1^ year^−1^, respectively, grazed from 6 a.m. to 6 p.m. from June to November each year. Each plot covered an area of 4.4 ha and was replicated three times.

In May 2020, we selected the heavy grazing plots as the experimental area of degraded grassland (CK). In each plot, two 10 m × 10 m fences were set up for grazer exclosure treatment (natural restoration, GE); at the same time, traditional open-top chambers (OTC) were deployed in each of the grazer exclosure areas to simulate warming (GE + W). These chambers, surrounded by Plexiglas fiberboards, featured a bottom area of 1.50 m^2^, a height of 0.51 m, and a top opening area of 0.79 m^2^. By 2023, the OTCs had effectively increased the mean temperature of the topsoil (0–10 cm) by 1.5°C.

### 2.2 Soil sampling and assaying

In mid-August 2023, topsoil samples were collected from each experimental plot (CK, GE, and GE + W) using the 5-point sampling method, totaling 18 soil samples (3 treatments × 3 blocks × 2 fences) = 18 soil samples. The soil samples were further divided into two, one of which was stored at −80°C for DNA extraction and amplicon sequencing, and the other immediately transported to the laboratory in a cooler for the determination of soil physicochemical properties.

The dichromate oxidation method was used to determine soil organic carbon (SOC) content, and a flow analyzer (AA3, SEAL Analytical, Germany) was used to determine ammonium nitrogen (NH_4_^+^-N) and nitrate nitrogen (NO_3_^−^-N) contents (Nelson and Sommers, 2018). Finally, soil pH was measured by a pH meter (BPH-7100, BELL Analytical Instruments, Dalian, China).

### 2.3 Soil microbial diversity assaying

Total soil DNA was extracted from fresh soil samples using the PowerSoil DNA Isolation Kit (MO BIO Laboratories, Carlsbad, United States). A NanoDrop UV–Vis spectrophotometer (ND-2000c, Nano Technologies, DE, United States) was used to measure the concentration and purity of soil DNA. The primers 515-forward (5′-GTGCCAGCMGCCGCGGTAA-3′) and 806-reverse (5′-GGACTACHVGGGTWTCTAAT-3′) were used to amplify bacterial 16S rDNA. The fungal primer sets ITS5-1737-forward and ITS2-2043-reverse were used to amplify the ITS1 variable region. Libraries were constructed using the TruSeq^®^ DNA PCR-Free Sample Preparation Kit. Sequencing was performed by Novogold Bioinformatics Ltd., Beijing, China. After training the naïve Bayes classifier based on the primer sequences, the ASVs were taxonomically annotated using the feature-classifier module based on the SILVA database release 138 (Liu D. et al., 2024).

### 2.4 Microbial network analysis

First, raw microbial ASVs were screened to exclude those with less than 15 occurrences in the 18 soil samples, where the number of ASVs represents microbial richness. Then, the microbial co-occurrence network was constructed in R software with the “Hmisc” package (R Core Development Team, R Foundation for Statistical Computing, Vienna, Austria), and sub-network parameters for each sample were extracted using the “igraph” package, including the numbers of nodes, edges, and network complexity. Network parameters were visualized using Gephi 0.10.1 software.^1^ The stability of the microbial network was quantified using the ratio of negative to positive cohesion, which represents the competitive and cooperative interactions between species in the community, respectively (Faust and Raes, 2012).


$$\text{cohesion}=\sum_{i=1}^{n}r_{i}\times c_{i}$$

where *n* is the number of taxa in the community, and *r_i_* and *c_i_* are the abundance and connectivity of taxa, respectively.

### 2.5 Statistical analysis

Significant differences in microbial diversity, composition, and network parameters between treatments were detected using one-way ANOVA and Duncan’s test with *p* = 0.05 indicating significance. The results were visualized using OriginLab 2022 software. The Shapiro–Wilk test was used to test for normality prior to the ANOVA. Treatment effects on microbial community composition were assessed using non-metric multidimensional scaling (NMDS) analysis of different distance matrices using the “vegan” package. The degree and significance of each treatment on microbial community structure were quantified by analysis of similarities (ANOSIM), and significance was verified by the dispersion test. Then, correlations between soil physicochemical factors and microbial diversity, composition, and network parameters were assessed using Pearson’s correlation. To better determine which soil properties were correlated with microbial composition, we performed redundancy analyses (RDA) using Canoco 5.0 software (Ithaca, NY, United States). We used a linear fit of prominent soil impact factors and the relative abundance of microbial dominant species using OriginLab 2022 software.

## 3 Results

### 3.1 Variation in soil microbial communities

**Figure 1:**
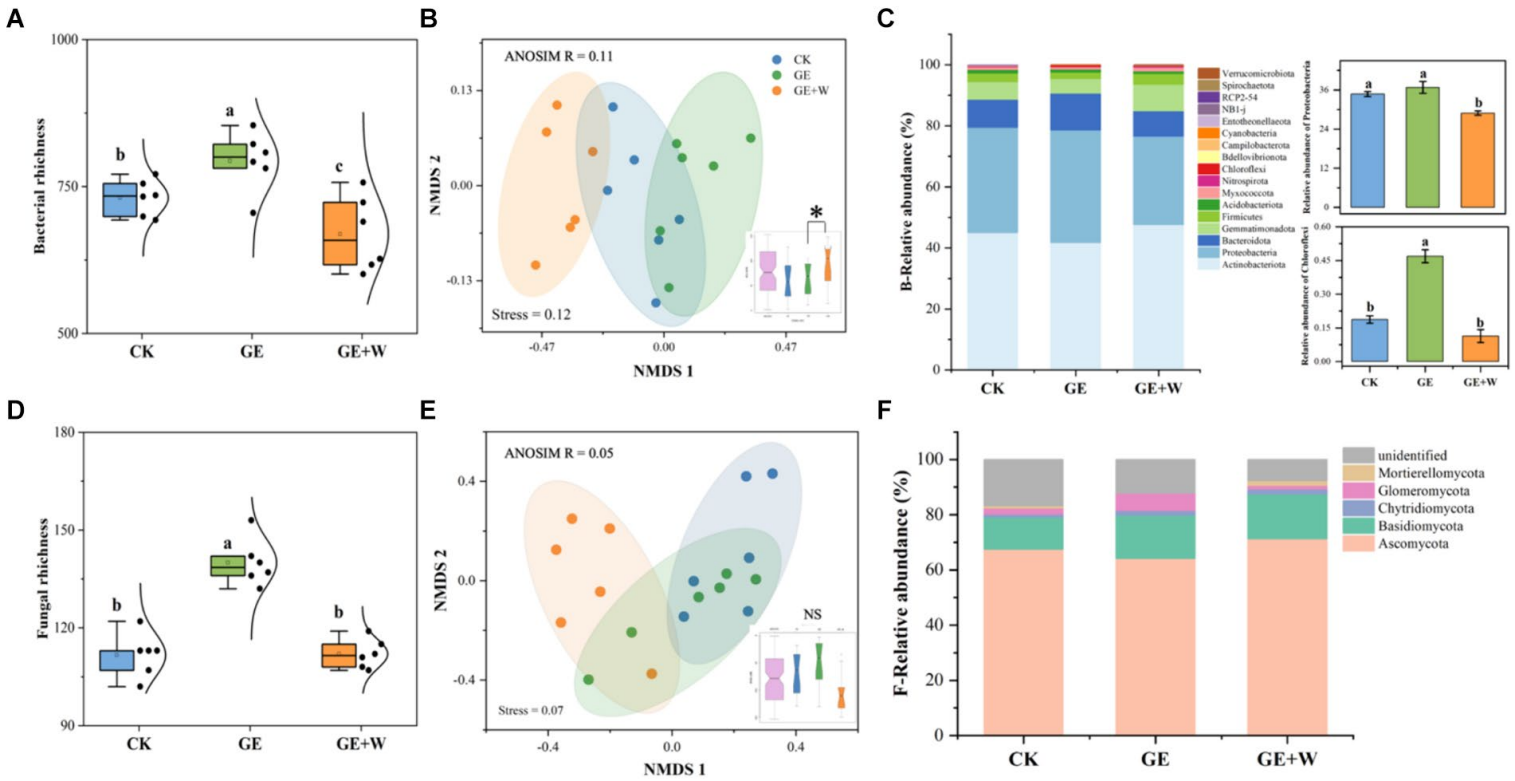
Variations in soil bacterial and fungal richness and abundance under different treatments (mean ± SE). **(A)** Bacterial richness, **(B)** bacterial beta-diversity, **(C)** bacterial phyla relative abundance, **(D)** fungal richness, **(E)** fungal beta-diversity, and **(F)** fungal phyla relative abundance. The lowercase letters indicate significant differences between means at *p* < 0.05 after *post-hoc* comparisons using Duncan’s test. CK, heavy grazing; GE, grazer exclosure; GE + W, grazer exclosure plus warming by 1.5°C.

Compared to CK, the GE treatment increased bacterial and fungal richness by 63 and 28, respectively, and the GE + W treatment significantly decreased bacterial richness by 62 (*p* < 0.05, Figures 1A,D). The NMDS analyses showed that bacterial communities differed significantly in ordinal spatial clustering, while fungal communities did not show significant differences (Figures 1B,E). The ANOSIM analysis further confirmed this result. Bacterial variability was significantly higher in the GE + W group than in the GE treatments (*R*^2^ = 0.11, Figure 1B), whereas fungal community variability under each treatment was not significant differences (Figure 1E).

The dominant bacterial phyla in each treatment were Actinobacteria, Proteobacteria, and Bacteroidota, with the GE + W treatment significantly decreasing the relative abundance of Proteobacteria compared to CK and the GE treatment increasing the relative abundance of Chloroflexi (*p* < 0.05, Figure 1C). Ascomycota and Basidiomycota were the dominant fungal phyla, and the fungal relative abundance of fungal species was not significantly different among the treatments (*p* > 0.05, Figure 1F).

### 3.2 Co-occurrence networks of microbial communities

**Figure 2:**
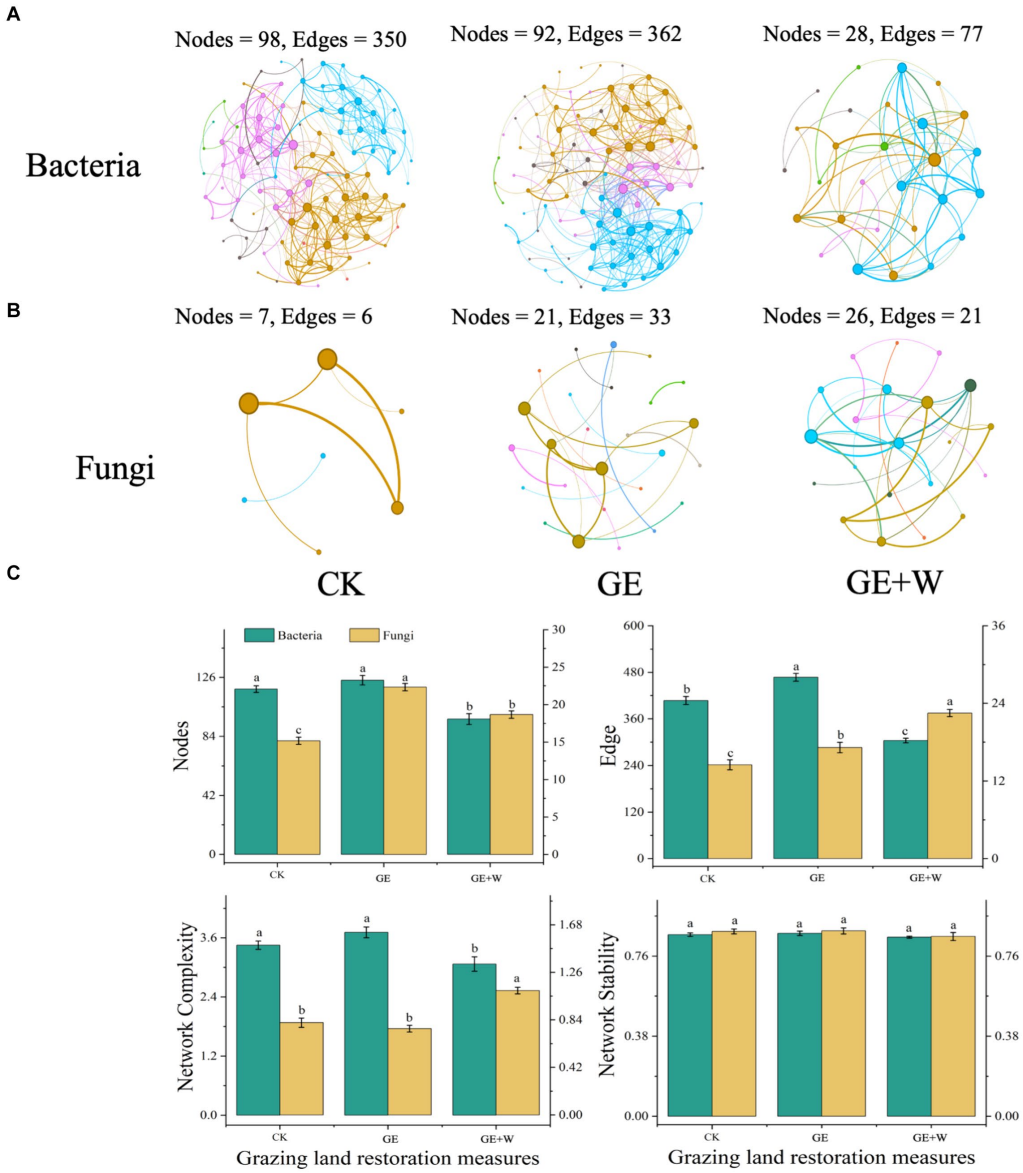
Network co-occurrence, complexity, and stability of soil bacterial and fungal communities. **(A)** Bacterial co-occurrence network, **(B)** fungal co-occurrence network, and **(C)** summary of bacterial and fungal network nodes, edges, network complexity, and network stability. The lowercase letters indicate significant differences between means at *p* < 0.05 after *post-hoc* comparisons using Duncan’s test. CK, heavy grazing; GE, grazer exclosure; GE + W, grazer exclosure plus warming by 1.5°C.

Soil microbial co-occurrence networks were constructed based on Spearman’s correlation between ASVs to explore interconnections between microbes after short-term grazer exclosure (Figures 2A,B). Overall, the GE + W treatment reduced the number of nodes and edges of the bacterial network compared to the CK treatment, and the degree of the network was reduced by 22.15 and 33.84%, respectively. The GE + W treatment also reduced the bacterial network complexity without changing stability (Figure 2C). The GE treatment increased the number of bacterial network edges relative to the CK control, but other properties were not changed. Compared with CK treatment, GE and GE + W treatments increased the number of fungal nodes and edges, but GE treatment did not affect fungal network complexity, while GE + W increased it (Figure 2B).

### 3.3 Relationship between the microbial community and soil properties

**Figure 3:**
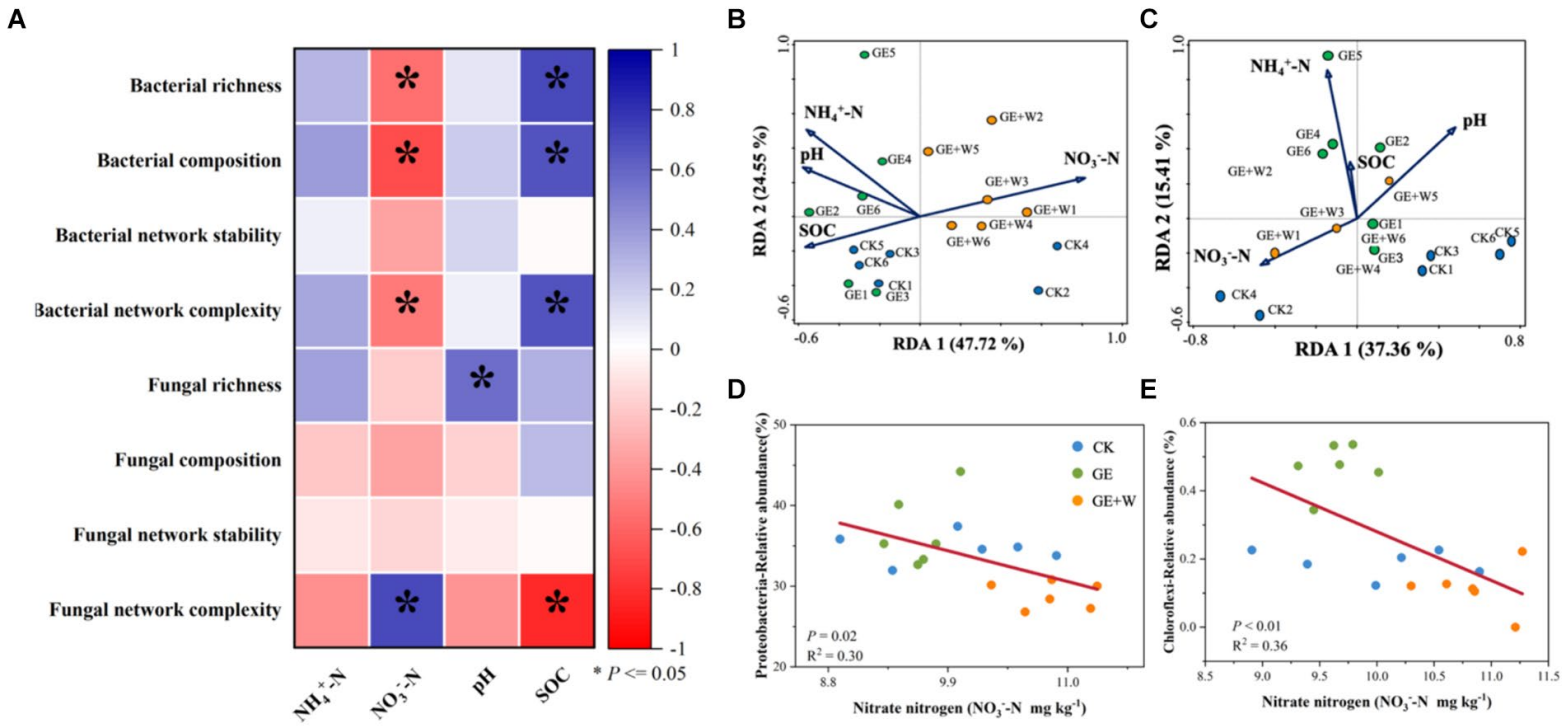
Relationships between microbial diversity, composition, network parameters, and soil properties. **(A)** Pearson correlation analysis of soil bacterial and fungal properties with soil properties. **(B)** Redundancy analysis of soil bacterial composition and soil properties. **(C)** Redundancy analysis of soil fungal composition and soil properties. **(D)** Linear fitting of soil nitrate nitrogen to Proteobacteria. **(E)** Linear fitting of soil nitrate nitrogen to Chloroflexi. NH_4_^+^-N: ammonium nitrogen, NO_3_^—^N, nitrate nitrogen; SOC, soil organic carbon. The blue color indicates a positive correlation, and the red color indicates a negative correlation; *p < 0.05.

Compared to the CK group, the GE treatment increased NH_4_^+^-N contents (*p* < 0.05), the GE + W treatment significantly increased NH_4_^+^-N, and NO_3_^−^-N contents (*p* < 0.05), but other soil properties did not differ significantly between treatments (Table 1). The soil content of NO_3_^−^-N was negatively correlated with bacterial richness, composition, and bacterial network complexity, and positively correlated with fungal network complexity, while SOC showed the opposite trend for each of these variables (Figure 3A; Supplementary Table S1).

**Table 1:** Variations in soil microbial diversity under different treatments (mean ± standard error).

	CK	GE	GE + W
Organic carbon (g kg^−1^)	15.19 ± 0.01 a	15.20 ± 0.01 a	15.16 ± 0.01 a
Ammonium nitrogen (mg kg^−1^)	4.46 ± 0.02 c	5.97 ± 0.16 a	4.78 ± 0.09 b
Nitrate nitrogen (mg kg^−1^)	9.99 ± 0.30 b	9.64 ± 0.10 b	10.85 ± 0.15 a
pH	7.89 ± 0.07 a	7.88 ± 0.03 a	7.94 ± 0.03 a

Different lowercase letters indicate significant differences between different treatments at *p* < 0.05. CK, heavy grazing; GE, grazer exclosure; GE + W, grazer exclosure plus warming by 1.5°C.

The RDA of microbial community composition with soil environmental factors showed that bacterial community composition on axis 1 showed differences with decreasing soil organic carbon and increasing nitrate nitrogen content (Figure 3B), where soil nitrate nitrogen content was the main influence factor influencing bacterial community composition (*p* < 0.05, Supplementary Table S2). Soil fungal community composition was not affected by environmental factors (Figure 3C). The relative abundance of both Proteobacteria (Figure 3D) and Chloroflexi (Figure 3E) declined with increasing nitrate nitrogen content.

## 4 Discussion

### 4.1 Grazer exclosure increases microbial richness without affecting network complexity, but this effect is impaired during warming

Consistent with hypothesis 1 and previous findings (Ding et al., 2019; Eldridge et al., 2017; Zhang M. X. et al., 2023), our results demonstrate that grazer exclosure treatment increased soil bacterial and fungal richness and altered the soil microbial community structure. Prior research by Shu et al. (2024) and Yang et al. (2022) supports the notion that grazer exclosure enhances soil microbial community structure and function by mitigating the disturbances caused by grazing. Similarly, Geng et al. (2023) found that grazer exclosure in desert steppe regions increased the relative abundance of key microbial taxa such as Actinobacteria, Proteobacteria, and Chloroflexi, thereby enhancing overall soil microbial diversity and metabolic activity. In our study, we observed a significant increase in the relative abundance of Chloroflexi, while the relative abundance of many dominant taxa did not change significantly during the 3-year restoration period. This finding contrasts with Yao et al. (2018), who reported a decrease in the relative abundance of Chloroflexi after the restoration of degraded desert steppe via fencing. This discrepancy may be attributed to the longer duration of exclosure in their study, as long-term grazing exclosure likely leads to an increase in soil nutrients, which is detrimental to the survival of oligotrophic bacterial communities. Concurrently, the increased nutrient availability enhances the reproductive rate and resource competitiveness of eutrophic bacteria, placing additional competitive pressure on oligotrophic bacteria and further reducing their diversity and relative abundance (Cheng et al., 2016; Cao et al., 2022; Chen et al., 2023). Moreover, the benefits of grazer exclosure on soil microbial communities can be modulated by other environmental factors, such as climate warming (Li et al., 2016; Guo et al., 2018, 2019). For example, the positive effect of exclosure on bacterial α-diversity is weakened by warming (Wu L. et al., 2022; He et al., 2022). Our findings corroborate this, as we observed that grazer exclosure plus warming treatment had a negative impact on bacterial richness. This is likely because increased temperatures exacerbate the metabolic costs for soil bacteria, leading to competitive displacement and reduced survival rates of temperature-sensitive species (Söllinger et al., 2022) such as Proteobacteria (Zhang et al., 2015; Zhao et al., 2024). Similarly, we found that warming treatment of exclosure areas reduced the relative abundance of Proteobacteria but did not affect the structure and composition of the fungal community. Fungi are more resistant to environmental changes and disturbances than bacteria because of their active dispersal traits (Roper et al., 2010; Liu Y. et al., 2024).

Our results demonstrate that grazer exclosure without warming treatment increased the number of soil microbial network nodes and edges but did not change bacterial network complexity or stability. In contrast, grazer exclosure with warming treatment had the same effect on the number of microbial network nodes, edges, and stability, but significantly decreased bacterial network complexity and increased fungal network complexity. These results suggest that soil networks become more connected as natural restoration proceeds (Morriën et al., 2017), whereas grazer exclosure under climate warming enhances the competitive advantage of fungi and adversely affects the stability of bacterial communities (Jia et al., 2023; Zhou et al., 2023; Zhu et al., 2023). Our results are consistent with previous research conducted in coastal areas (Zhou et al., 2021) and the Loess Plateau (Wang et al., 2024), which suggests that warming leads to bacterial community instability due to shrinking soil microbial ecological niches (Yang et al., 2013; Wu et al., 2021). However, studies conducted in the tallgrass prairie (Yuan et al., 2021) and the Tibetan Plateau (Chen et al., 2023) found that warming enhances the complexity and stability of bacterial networks. These inconsistent observations may result from differences in ecosystem vulnerability and sensitivity (Zhang M. et al., 2023). The desert steppe responds strongly to climate change due to its arid climate, infertile soils, and ecological fragility (Yang et al., 2020); in which case, microbial networks may loosen or even collapse with warming (Ullah et al., 2018).

### 4.2 Grazer exclosure impacts bacterial microbial communities by influencing nitrate nitrogen content under warming

As expected, our experiment revealed that soil nitrate nitrogen content influenced microbial community composition, evidenced by a decrease in the relative abundance of soil metazoans and green curvilinear bacteria with increasing nitrate nitrogen content. We found that grazer exclosure treatment increased soil ammonium nitrogen content, while grazer exclosure plus warming increased nitrate nitrogen content, in line with previous studies indicating that grazer exclosure would allow soil nutrients to recover (Cheng et al., 2016; Wang et al., 2019; Liu et al., 2019). Climate warming affects soil nitrogen availability and subsequently impacts the microbial community (Li et al., 2024; Bai et al., 2013). As temperatures rise, microbial activity and metabolism increase, stimulating the mineralization of organic nitrogen compounds into inorganic forms such as ammonium (NH₄^+^) and nitrate (NO₃^−^), which are more readily available to microorganisms (Liu et al., 2022). Additionally, climate warming enhances nitrification in grassland soils, accelerating the conversion of ammonium to nitrate. Higher nitrate nitrogen content consequently inhibits microbes with lower nitrogen requirements (Shi et al., 2023). Zhou et al. (2015) also found that soil nitrate nitrogen content has a major influence on microbial community structure, with high nitrate nitrogen content resulting in lower Pedosphaerae abundance. We also observed that soil nitrate nitrogen content was negatively correlated with bacterial richness and network complexity and had a negative effect on bacterial community composition. In contrast, there was a positive correlation with fungal complexity and no significant correlation to fungal community composition and diversity. Indeed, bacterial community composition is primarily regulated by soil factors, whereas fungal community composition is regulated by plants (Su et al., 2023). A recent meta-analysis showed that shifts in microbial α-diversity under global change were mainly explained by soil pH (Zhou et al., 2020). Kang et al. (2021) also found changes in soil microbial diversity and community structure were caused by pH and organic carbon content. This is inconsistent with our findings in which soil pH and organic carbon content were not found to affect the microbial community, which might be due to a shorter period of grazer exclosure in our study.

## 5 Conclusion

Overall, we showed that grazer exclosure is effective in increasing soil microbial diversity without affecting the stability of their networks. These benefits may be affected by climate warming, which reduces bacterial diversity and network complexity by increasing nitrate nitrogen contents. Our study demonstrated that the recovery of soil microbial communities in degraded grasslands through grazing exclosure may be slow under future warming scenarios. Moreover, long-term grazing exclosure may reduce the income of herders and, consequently, regional and national economies. Therefore, land managers need to consider the environmental as well as social and economic implications of degraded grassland restoration measures. Developing climate change-adapted grassland management measures, such as rotational grazing, could be a suitable option for maintaining grassland sustainability.

## Data availability statement

The original contributions presented in the study are included in the article/Supplementary material, further inquiries can be directed to the corresponding author.

## Author contributions

JQ: Conceptualization, Data curation, Formal analysis, Methodology, Software, Visualization, Writing – original draft, Writing – review & editing. JZ: Data curation, Software, Visualization, Writing – review & editing. SL: Data curation, Software, Visualization, Writing – review & editing. FZ: Data curation, Writing – review & editing. BZ: Writing – review & editing. MZ: Funding acquisition, Supervision, Writing – review & editing.

## Footnotes

^1^ http://gephi.github.io/
