# Supplementary material for: Impact of climate warming on soil microbial communities during the restoration of the inner Mongolian desert steppe
Source: Front Microbiol. 2024 Sep 6;15:1458777. doi: 10.3389/fmicb.2024.1458777 (PMC11412859; doi:10.3389/fmicb.2024.1458777)
Supplement: Supplementary file 1 [file Table_1.DOCX]

Supplementary Material

# Supplementary Table 1 Pearson’s correlation between soil properties and microbial richness, composition, network stability, and complexity. NH_4_^+^-N: Ammonium nitrogen, NO_3_^-^-N: Nitrate nitrogen, and SOC: soil organic carbon. * indicates significant correlations (*P* < 0.05) between pairs of independent index.

|  | NH_4_^+^-N | NO_3_^-^-N | pH | SOC | Bacterial richness | Bacterial composition | Bacterial network stability | Bacterial network complexity | Fungal richness | Fungal composition | Fungal network stability | Fungal network complexity |
| --- | --- | --- | --- | --- | --- | --- | --- | --- | --- | --- | --- | --- |
| NH_4_^+^-N | 1 |  |  |  |  |  |  |  |  |  |  |  |
| NO_3_^-^-N | -0.18 | 1 |  |  |  |  |  |  |  |  |  |  |
| pH | 0.51 * | -0.07 | 1 |  |  |  |  |  |  |  |  |  |
| SOC | 0.54 * | -0.50 * | 0.22 | 1 |  |  |  |  |  |  |  |  |
| Bacterial richness | 0.29 | -0.54 * | 0.12 | 0.72 * | 1 |  |  |  |  |  |  |  |
| Bacterial composition | 0.4 | -0.68 * | 0.20 | 0.67 * | 0.56 * | 1 |  |  |  |  |  |  |
| Bacterial network stability | 0.07 | -0.35 | 0.18 | -0.02 | 0.13 | 0.38 | 1 |  |  |  |  |  |
| Bacterial network complexity | 0.35 | -0.52 * | 0.07 | 0.68 * | 0.58 * | 0.62 * | 0.36 | 1 |  |  |  |  |
| Fungal richness | 0.37 | -0.20 | 0.58 * | 0.31 | 0.38 | 0.43 | 0.10 | 0.35 | 1 |  |  |  |
| Fungal composition | -0.21 | -0.35 | -0.17 | 0.27 | 0.30 | 0.46 | 0.46 | 0.44 | 0.01 | 1 |  |  |
| Fungal network stability | -0.09 | -0.14 | -0.08 | -0.02 | 0.31 | 0.007 | 0.24 | 0.30 | 0.19 | 0.43 | 1 |  |
| Fungal network complexity | -0.15 | 0.70 * | 0.004 | -0.67 * | -0.68 * | -0.84 * | -0.09 | -0.61 * | -0.36 | -0.36 | 0.05 | 1 |

# Supplementary Table 2 Redundancy analysis of soil bacterial and fungal composition and soil properties.

|  | Factor | Organic carbon (g kg^-1^) | Ammonium nitrogen (mg kg^-1^) | Nitrate nitrogen (mg kg^-1^) | pH |
| --- | --- | --- | --- | --- | --- |
| Bacteria | Explains (%) | 3.8 | 3.3 | 12.9 | 81 |
|  | Contribution (%) | 13.7 | 11.7 | 46 | 28.7 |
|  | F-value | 0.7 | 0.6 | 2.4 | 1.5 |
|  | *P*-value | 0.71 | 0.85 | 0.03 | 0.14 |
| Fungi | Explains (%) | 4.5 | 5.2 | 5.2 | 6 |
|  | Contribution (%) | 21.4 | 24.9 | 25.1 | 28.6 |
|  | F-value | 0.7 | 0.9 | 0.9 | 1.0 |
|  | *P*-value | 0.77 | 0.67 | 0.61 | 0.49 |
